# Computational Predictions of Volatile Anesthetic Interactions with the Microtubule Cytoskeleton: Implications for Side Effects of General Anesthesia

**DOI:** 10.1371/journal.pone.0037251

**Published:** 2012-06-25

**Authors:** Travis J. A. Craddock, Marc St. George, Holly Freedman, Khaled H. Barakat, Sambasivarao Damaraju, Stuart Hameroff, Jack A. Tuszynski

**Affiliations:** 1 Department of Physics, University of Alberta, Edmonton, Alberta, Canada; 2 Department of Laboratory Medicine and Pathology, University of Alberta, Edmonton, Alberta, Canada; 3 Center of Marine Sciences, Foundation for Science and Technology, University of Algarve, Campus Gambelas, Faro, Portugal; 4 Departments of Anesthesiology and Psychology, Center for Consciousness Studies, The University of Arizona Health Sciences Center, Tucson, Arizona, United States of America; 5 Department of Oncology, Cross Cancer Institute, University of Alberta, Edmonton, Alberta, Canada; Emory University, United States of America

## Abstract

The cytoskeleton is essential to cell morphology, cargo trafficking, and cell division. As the neuronal cytoskeleton is extremely complex, it is no wonder that a startling number of neurodegenerative disorders (including but not limited to Alzheimer’s disease, Parkinson’s disease and Huntington’s disease) share the common feature of a dysfunctional neuronal cytoskeleton. Recently, concern has been raised about a possible link between anesthesia, post-operative cognitive dysfunction, and the exacerbation of neurodegenerative disorders. Experimental investigations suggest that anesthetics bind to and affect cytoskeletal microtubules, and that anesthesia-related cognitive dysfunction involves microtubule instability, hyper-phosphorylation of the microtubule-associated protein tau, and tau separation from microtubules. However, exact mechanisms are yet to be identified. In this paper the interaction of anesthetics with the microtubule subunit protein tubulin is investigated using computer-modeling methods. Homology modeling, molecular dynamics simulations and surface geometry techniques were used to determine putative binding sites for volatile anesthetics on tubulin. This was followed by free energy based docking calculations for halothane (2-bromo-2-chloro-1,1,1-trifluoroethane) on the tubulin body, and C-terminal regions for specific tubulin isotypes. Locations of the putative binding sites, halothane binding energies and the relation to cytoskeleton function are reported in this paper.

## Introduction

Despite the extensive electrophysiological studies regarding the effects of inhaled anesthetics on membrane ion channels and receptor proteins [1]–[3] the exact molecular mode of action of anesthetics remains uncertain. In addition to altering the function of membrane ion channels and receptors *in vitro* [4]–[14], the inhaled anesthetics are known to affect enzymes [15]–[17] as well as many cytoplasmic proteins in the mammalian central nervous system [18]–[22], providing multiple targets for their actions including side effects. Among these cytoplasmic proteins is tubulin, the component protein of cytoskeletal microtubules.

Tubulin proteins polymerize to form microtubules (MTs), nanoscale cylindrically shaped protein polymers that are part of the cellular cytoskeleton. The neuronal MT cytoskeleton, in particular, possesses a unique architecture [23], responsible for maintaining highly asymmetric neuron morphology and the intracellular transport of vesicles. Unlike MTs in all other cells, the MTs in dendrites are interrupted and oriented in local networks of mixed polarity. Moreover, the ionotropic GABA_A_ receptor is anchored to the MT cytoskeleton via associated proteins [24], and an intact MT structure has been shown to be essential for the activity of GABA_A_ receptors [25]. Likewise, MTs have been shown to modulate both sodium and calcium current within neurons [26]–[30].

A large number of neurological brain disorders bear the common feature of some kind of disruption to the neuronal cytoskeleton, particularly MTs, either directly or indirectly through associated proteins. These include neurodegenerative disorders such as Alzheimer’s disease, Parkinson’s disease, and Huntington’s disease [31]. The relation between anesthesia, postoperative cognitive decline and dementia (in Alzheimer’s disease and the other neurodegenerative disorders) is uncertain [32]–[35]. Anesthesia may exacerbate neurodegeneration, and/or have its own deleterious effect. Cytoskeletal MTs are a common link, affected by anesthetics and disrupted in neurodegeneration.

This suggests an important role for the MT cytoskeleton in anesthetic action, with potential side effects related to post-operative cognitive dysfunction. Recently it has been shown that genetic expression of tubulin is altered following exposure to desflurane [36], sevoflurane [37], halothane, and isoflurane [38]. LeFreche et al. [39], [40] further showed that cognitive dysfunction following sevoflurane anesthesia was associated with hyper-phosphorylation of the MT-associated protein (MAP) tau, separation of tau from MTs, and MT instability, the same signs associated with neurofibrillary tangles (NFTs) in Alzheimer’s disease. Anesthetic binding to tubulin in MTs may be involved in anesthetic action and toxicity.

**Figure 1:**
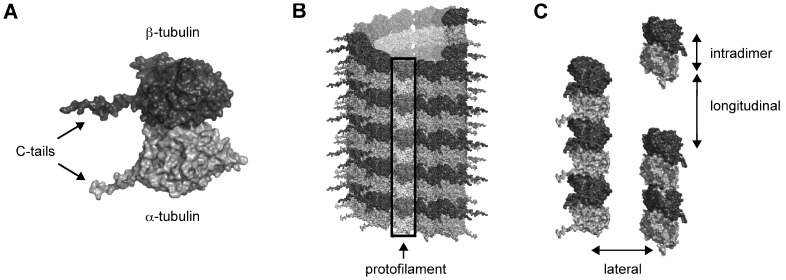
Tubulin in MT formation. (A) Tubulin dimer. Light grey –α-tubulin, Dark Grey – β-tubulin. C-terminal tails extend from the main tubulin body. (B) B-lattice MT with protofilament highlighted. (C) Tubulin interactions in MT formation. Intradimer – between α- and β-tubulins, Longitudinal – between dimers in a protofilament, Lateral – between protofilaments.

Tubulin, a peanut-shaped heterodimer with α and β monomers, has been identified as a direct binding target for halothane [22]. Additionally, experiment shows volatile anesthetics, particularly halothane, alter the tubulin self-assembly rates into MTs in a number of systems, both *in vivo* [41]–[46] and *in vitro* [47], albeit at extremely high concentrations. When polymerized into MT form, each tubulin subunit interacts with surrounding dimers, forming longitudinal contacts between dimers along the protofilament length, and lateral contacts between protofilaments (see Figure 1). There also exists an intradimer interaction between the α- and β- monomers of a single tubulin dimer. In the more prevalent B-lattice MT formation the lateral contacts are formed between like subunits (i.e. α-monomer to α-monomer, and β-monomer to β-monomer on adjacent protofilaments). In the less common A-lattice, lateral interactions are between α and β monomers. Volatile anesthetics may inhibit MT assembly dynamics by a direct molecular interaction between the anesthetic molecule and the tubulin dimer hindering dimer-dimer, or intradimer interactions. It is also feasible that volatile anesthetics exert their action on MT dynamics through an alteration of the local environment affecting the highly flexible C-terminal tail regions of tubulin. Clearly, the site and mechanism of anesthetic action on MT assembly and stability remain to be determined.

Experimental methods to study anesthetic binding to proteins include NMR spectroscopy, photoaffinity labeling, and site directed mutagensis. NMR and photoaffinity labeling techniques can only be applied to purified proteins available in relatively large quantity. Currently, NMR methods are only capable of determining the structure of protein complexes with masses up to 20–30 kDa, which is well below the 110 kDa size of the tubulin heterodimer, rendering this method not directly applicable to this problem [48]. Without *a priori* knowledge of putative binding sites, site directed mutagenesis is also hindered by protein size. Direct photoaffinity labeling with halothane requires combination with methods such as protein digestion followed by mass spectroscopy to determine binding location, which also benefits from previously predicted locations to determine experimental protocols.

Surface geometry techniques used to predict anesthetic binding sites on proteins are based on static structural data [49], [50]. These methods suffer a general weakness by not accounting for protein dynamics, neglecting rearrangements of local protein atoms and the resulting change in binding site availability. To address these issues we use a combination of molecular dynamics (MD) and surface geometry based binding site prediction to identify general putative volatile anesthetic binding sites on, or in, the tubulin protein. Blind docking followed site prediction to obtain halothane (2-bromo-2-chloro-1,1,1-trifluoroethane) binding energy estimates, as the majority of experiments between volatile anesthetics and tubulin investigate the interaction with halothane.

## Results

### Putative Volatile Anesthetic Binding Sites on the Tubulin Body

**Figure 2:**
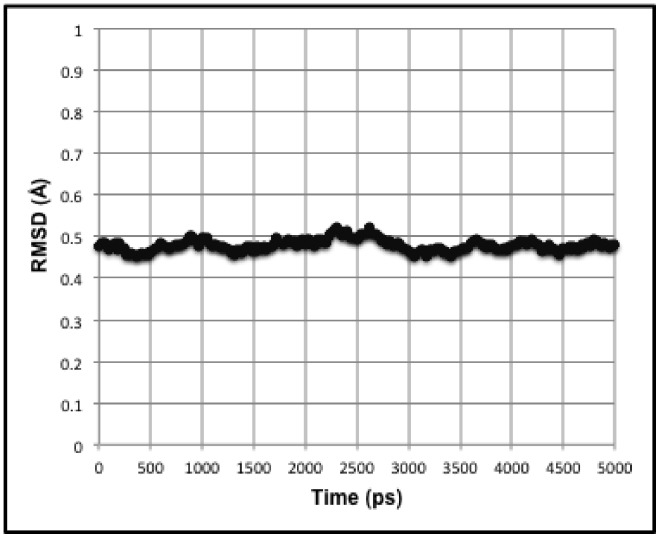
Plot of protein backbone RMSD over 5 ns simulation.

A short 5 ns MD simulation was performed on two tubulin dimers in MT geometry with periodic boundary conditions, effectively modeling two infinite protofilaments. This 5 ns simulation is too brief to be representative of the entire conformational ensemble. However, it does serve to allow side chain motion while keeping the protein backbone of the structure relatively stable (see Figure 2).

Clustering of the 5 ns MD simulation trajectories resulted in 11 distinct protein conformations, with each cluster containing several snapshots of the protein at different time steps. Taking each snapshot in a cluster to be represented by the average conformation of the cluster resulted in the 11 dominant conformations existing for various portions of the simulation (see Table 1). Since each dominant conformation is represented by the average conformation of several snapshots at different timesteps, a given dominant conformation represents a certain percentage of the MD simulation (see Table 1 - % Simulation).

**Table 1:** Percent of simulation for the dominant tubulin conformations.

TubulinConformation	Timesteps(out of 251)	Simulation %	Rank
1	13	5.18	6
2	2	0.80	2
3	29	11.55	4
4	9	3.59	7
5	57	22.71	2
6	32	12.75	3
7	69	27.49	1
8	4	1.59	8
9	13	5.18	6
10	19	7.57	5
11	4	1.59	8

A modified PASS algorithm [51] was performed on each of the dominant conformations for both dimers in MT protofilament geometry, and for each dimer separately. PASS predicts putative binding sites (hydrophobic crevices and pockets) through an iterative coating of the protein surface with probe spheres. Potential sites are based on the burial depth of these spheres. Modification of this algorithm to additionally measure for hydrophobicity yields efficient prediction of volatile anesthetic binding sites [49]. This procedure yielded numerous putative anesthetic-binding sites on tubulin, which would be valid for any volatile anesthetic.

**Figure 3:**
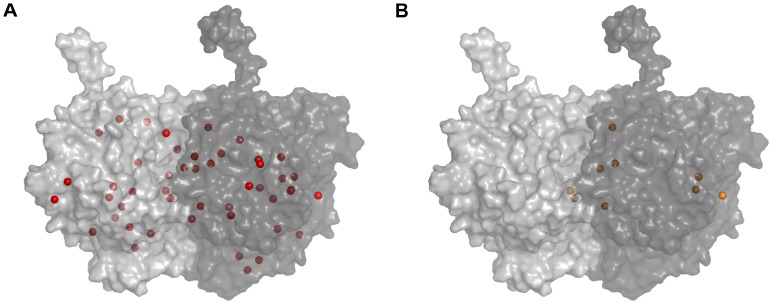
Putative volatile anesthetic binding sites on the tubulin body. (A) 47 total sites (red spheres) with persistence ranging from 0.80% to 100%. (B) 9 most persistent, and probable, sites (orange spheres), with persistence of 70% or greater.

Due to the motion of side chains the predicted sites varied between the different protein conformations. The DBSCAN method [52] spatially grouped the predicted sites yielding 47 unique potential binding sites on the tubulin protein, however some sites were not found in all of the conformations. As such, each site was assigned a persistence value denoting the percentage of the MD simulation in which the potential binding site was found. The persistence of each site was calculated by taking the sum of the simulation percentages (see Table 1 - % Simulation) for each of the dominant conformations on which the site was predicted (e.g. If a site was found for dominant tubulin conformation 1, 2, and 3, the persistence would be 5.18% + 0.80% + 11.55% = 17.53% of the MD simulation). Persistence values varied greatly from 0.80% to 100% of the 5 ns simulation (see Table 2 and 3). Of the 47 predicted sites, 9 persisted for more than 70% of the simulation, and of these 5 persisted for the entire simulation (see Figure 3).

**Table 2:** Persistence, surrounding residues, and halothane binding energies of putative volatile anesthetic binding sites on a single αβ-tubulin dimer.

Site	Persistence(Simulation %)	Residues within 5 Å^a^	Energy (kcal/mol)
α*-tubulin*			
23	100.00	αQ11, αA12	−2.54
8	51.00	αI188, αA421, αD424, αM425, αA426, αA427, αL428	−2.70
20	43.82	αG321, αP359, αP360, αT361, αV362, αV371, αQ372	−2.91
32	27.49	αY103, αY408, αE417, αF418	−3.39
25	21.51	αV62, αP63, αV66, αF67, αF87, αH88, αP89, αE90, αQ91	−3.31
2	20.32	αL23, αN228, αR229, αQ233, αP364	−2.67
27	11.55	αR123, αL132, αD160	−2.17
26	11.55	αS6, αH8, αC20, αR64, αA65, αL136, αV235, αS236	−2.90
3	6.77	αN216, αP274, αI276, αQ285, αL286, αI291, αN300	−2.46
22	5.18	αH107, αY108, αI115, αL152, αL153, αR156	−2.60
19	5.18	αV288, αA289, αV324, αK326, αD327	−2.10
29	3.59	αT292, αN293, αD327, αA330, αA331, αA334	−2.45
33	1.59	αH266, αM313, αA314, αN380, αT382, αY432	−2.89
35	1.59	αA174, αP175, αM203, αV204, αD205, αL269, αV303, αI384	−2.77
13	1.59	αG310, αM313, αT382, αA385, αE433	−2.52
24	0.80	αF202, αV204, αI209, αL230, αI231, αI234, αY272, αM302, αV303	−3.17
β*-tubulin*			
4	100.00	βV295, βF296, βV315, βA316, βA317, βM332	−3.12
21	100.00	βV171, βI204, βN206, βY210, βY224, βV231	−2.85
7	78.88	βT240, βC241, βL248, βN249, βA354, βV355	−3.13
1	70.12	βC12, βV171, βV172, βP173, βV177, βD179	−3.04
12	34.26	βY108, βV115, βL152, βL153, βK156, βI157	−2.99
30	31.87	βA208, βR215, βM301, βM302, βA304	−2.73
10	27.09	βL219, βT221, βP222, βT223, βL227, βL230	−2.68
18	23.51	βS117, βD120	−2.66
17	21.91	βV172, βP173, βS174, βP175, βC203, βD205, βA303, βL387, βI391	−2.89
34	15.94	βL313, βT314, βP348, βN350, βV351, βK352	−2.81
9	12.75	βR284, βL286, βT287, βL291, βK372, βM373	−2.79
6	11.55	βL119, βD120, βV122, βR123, βI157	−2.20
31	9.16	βA298, βK299, βM301, βM302, βA303, βA304	−2.85
16	7.57	βR123, βA126, βL132, βQ133, βF135, βY161, βR164	−2.52
11	1.59	βI31, βT33, βK60, βY61, βV62	−2.50
15	1.59	βL119, βD120, βV122, βR123, βF135, βI157	−2.48

a Residues are numbered according to the scheme of Löwe et al. [61].

**Table 3:** Persistence, surrounding residues, and halothane binding energies of putative volatile anesthetic binding sites at tubulin interfaces.

Site	Persistence(Simulation %)	Residues within 5 Å^a^	HalothaneBindingEnergy (kcal/mol)
*Intradimer*			
5	100.00	αQ11, αA12, αI171, αV177, αS178, αT179 / βQ247	−2.74
37	100.00	αM398 / βN258, βP261, βL313	−2.76
38	84.06	αA180, αV181/ βK254, βV257, βT314	−2.72
41	56.18	αF404 / βI165, βD199, βA256, βV257, βV260	−2.52
44	22.71	αT223 / βS324, βA354, βV355, βC356, βD357	−2.29
14	12.75	αH406 / βE196, βN197, βT198, βL273	−2.62
46	7.57	αQ176, αS178, αA180, αV181 / βK352	−2.62
40	0.80	αP184, αR390, αL391 / βI347	−2.52
*Longitudinal*			
39	88.05	αL242, αA247, αV250 / βQ13, βT145, βD179	−2.44
*Lateral -* α*-tubulins*			
43	26.29	αI212, αN216, αA273, αP274, αV275, αL286, αE290, αI291, αN300 / αK124	−2.33
36	19.52	αH283, αS287, αE290 / αE55, αV62, αE90	−2.68
*Lateral -* β*-tubulins*			
28	40.24	βD90, βV93, βF94 / βT252	−2.69
47	40.24	βS128 / βV288, βP289, βE327, βQ331	−2.35
45	7.57	βV62, βF87, βR88 / βT287, βV288, βM373	−2.84
42	0.80	βD90 / βI212, βR215, βT216, βL217, βP274, βK299	−2.69

a Residues are numbered according to the scheme of Löwe et al. [61].

### Halothane Docking

#### Tubulin body

**Figure 4:**
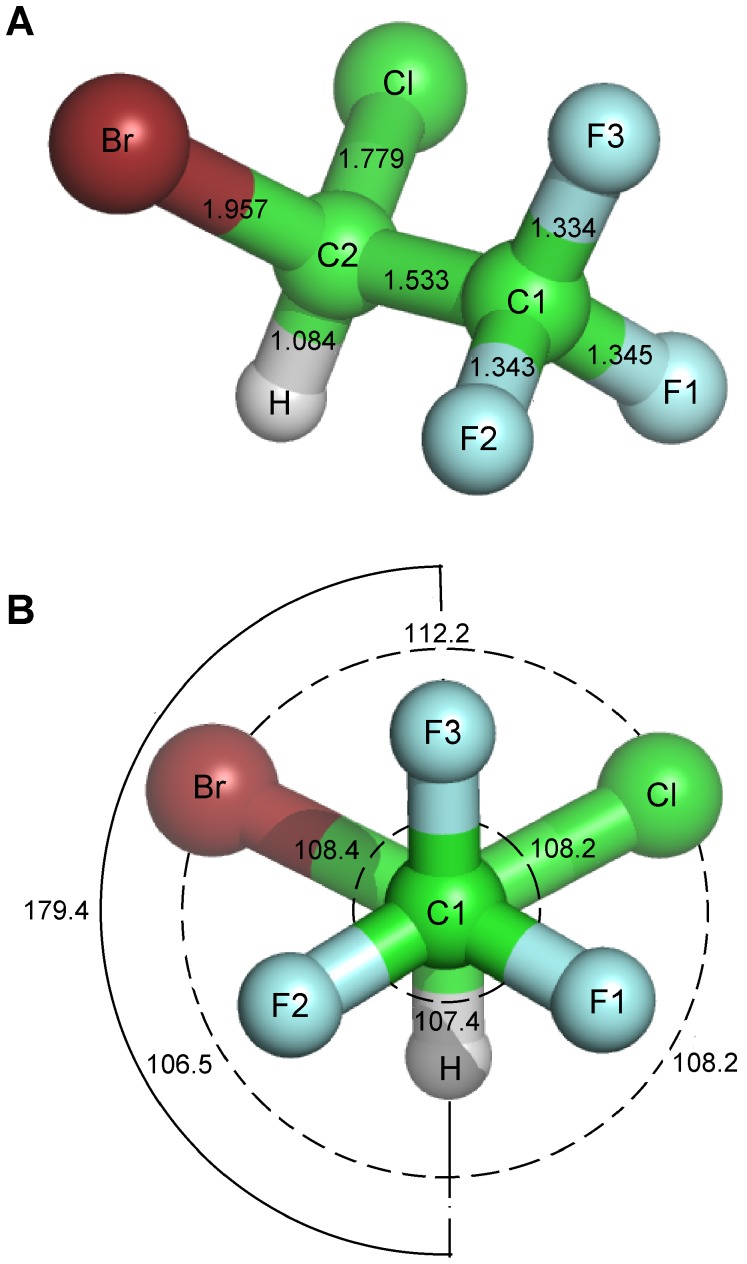
Halothane molecule structure parameters. (A) Bond lengths in Å. (B) Bond (dashed), and dihedral (solid) angles in degrees. Parameters obtained from an *ab initio* structure calculation [108].

Focused docking of a halothane molecule (see Figure 4) at each of the predicted putative volatile anesthetic binding sites on each of the tubulin clusters yielded varying binding energies between clusters for a given site. The binding mode with the lowest binding energy value for a given site was taken, generally resulting in one binding mode whose binding energy is listed in Table 2. In the case of multiple binding modes, the binding energy of the largest cluster was taken. Examination of the energy contributions yielded binding due mainly to van der Waals interactions with the Cl and Br atoms generally contributing the largest portion. Other energy contributions served to weaken this interaction.

#### C-terminal tails

**Figure 5:**
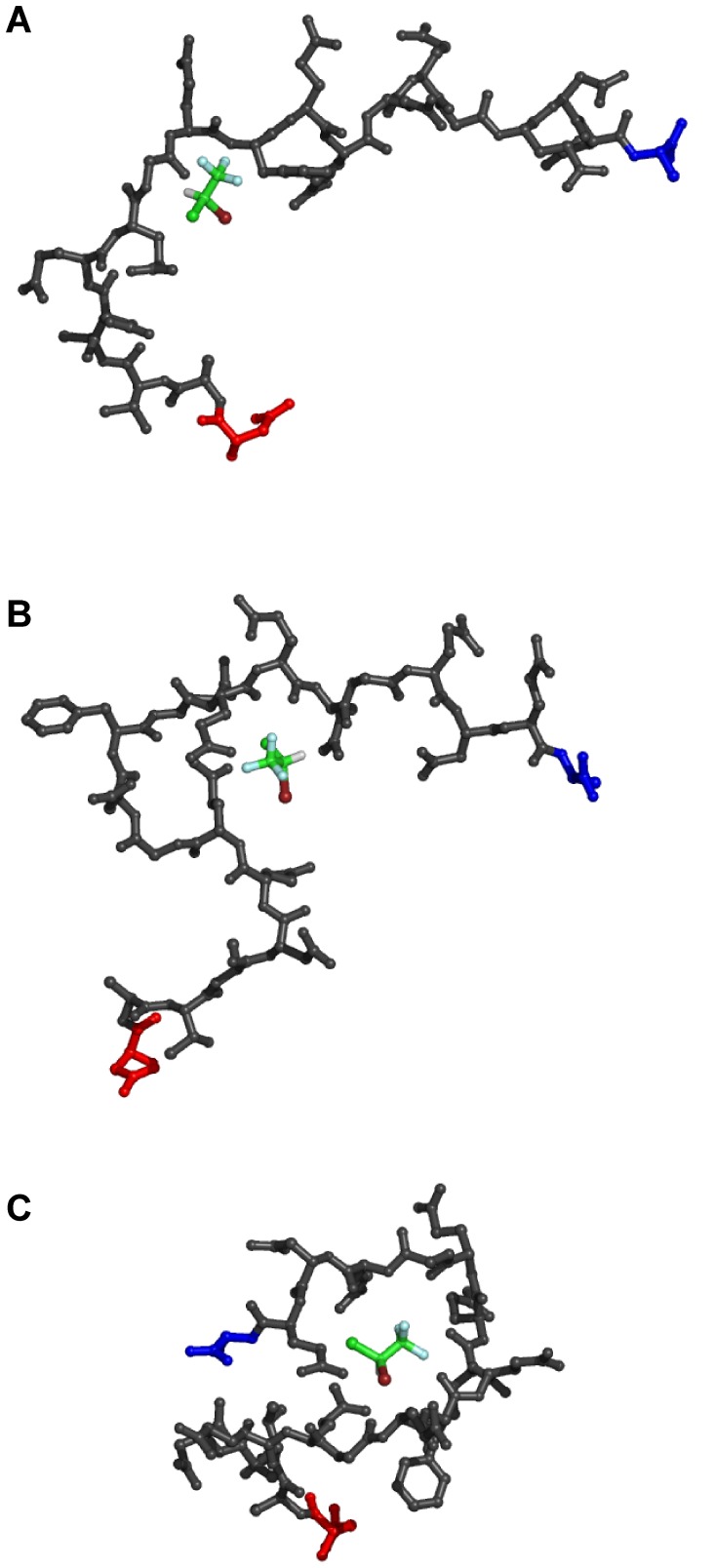
Representative halothane binding modes on the TUBB2B C-terminal tail. Red – N-terminal end connecting to the main tubulin body (body not shown for clarity), Blue – C-terminus. (A) −1.68 kcal/mol, (B) −2.3 kcal/mol, and (C) −2.79 kcal/mol.

For each tubulin isotype found in the brain, 50 distinct conformations of the C-terminal tail regions were generated sampling the tail conformational space (see Figure 5). Blind docking of the halothane molecule to each of the tail conformations resulted in various binding locations and in poses dependent on the sequence of the tail, as well as the specific tail conformation. The range of halothane binding energies for each of the tubulin isotypes is given in Table 4. The energy contributions yielded binding due mainly to van der Waals interactions again with the Cl and Br atoms contributing the largest portion. In general, binding energies increased with the number of available surrounding residues. Thus, tail conformations, which were compacted, forming loops or coils, provided more favorable binding conditions. Binding energies for these ideal binding-conditions were comparable to binding on the tubulin body.

**Table 4:** Tubulin isotype, sequence and halothane binding energy range for the C-terminal tail regions found in the brain.

Tubulin Isotype	C-terminal tail sequence^a^	Halothane Binding Energy Range (kcal/mol)
α-tubulin		
TUBA1A	DYEEVGVDSVEGEEEGEEY	−1.89 to −2.95
TUBA1C	DYEEVGADSADGEDEGEEY	−1.75 to −2.57
TUBA4A	DYEEVGIDSYEDEDEGEE	−1.86 to −2.78
β-tubulin		
TUBB	DATAEEEEDFGEEAEEEA	−1.60 to −2.82
TUBB2A / TUBB2B	DATADEQGEFEEEEGEDEA	−1.68 to −2.79
TUBB2C	DATAEEEGEFEEEAEEEVA	−1.62 to −3.12
TUBB3	DATAEEEGEMYEDDEEESEAQGPK	−1.63 to −2.81
TUBB4	DATAEQGEFEEEAEEEVA	−1.63 to −3.00

a Adapted from Luduena et al. [94].

### Microtubule Polymerization Assay

**Figure 6:**
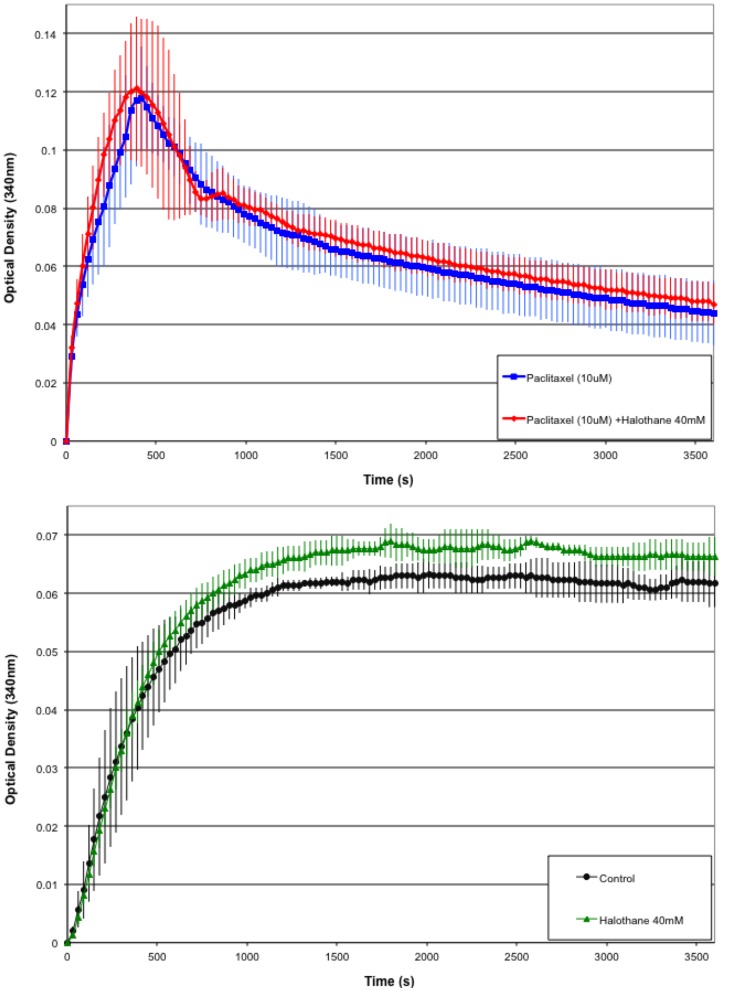
Microtubule polymerization assays. Black circle – General Tubulin Buffer (80 mM PIPES, MgCl_2_, 0.5 mM EGTA, pH 6.9). Green triangle – General Tubulin Buffer + 40 µM halothane. Blue square – General Tubulin Buffer + 10 µM paclitaxel. Red diamond – General Tubulin Buffer + 10 µM paclitaxel + 40 µM halothane. Mean values and standard deviation shown.

Multiple polymerization assays were run for tubulin alone (control), tubulin with 10 µM paclitaxel, tubulin with 40 mM halothane, and tubulin with both 10 µM paclitaxel and 40 mM halothane (see Figure 6). A 40 mM halothane concentration was used to ensure interaction of halothane with tubulin. The control run showed optical density results consistent with normal tubulin polymerization. In the presence of 10 µM paclitaxel optical density increases sharply and falling off in the longer time limit. This is consistent with normal results obtained from mist experiment with quick polymerization showing the stabilizing effect of paclitaxel. In the presence of both 10 µM paclitaxel and 40 mM halothane the optical density curve is comparable to that of paclitaxel alone within standard error. This indicates that halothane has no effect on the interaction between paclitaxel and tubulin. In the presence of 40 mM halothane alone the optical density curve resembles the control situation, however the standard error indicates difference between the curves with a small increase in the optical density.

## Discussion

### Interaction Mechanisms

The average brain volume is 1.4 L. Assuming approximately 10^9^ tubulin dimers per neuron, with 10^11^ neurons per brain, the average concentration of tubulin protein in the brain is 120 µM. However, in the neuron this may be 2–3 times higher since glial cells, possessing a MT density much less than neurons, are considered to comprise 50% of the brain. The remaining portion is composed of both neurons and other necessary structures such as ventricles, blood vessels etc. Comparing an assumed total neuronal tubulin protein concentration of 240–360 µM, to a halothane concentration of 250–500 µM (∼1 to 2 MAC) gives 1 to 2 halothane molecules per tubulin dimer at clinically relevant concentrations. However, occupancy depends not on equilibrated concentrations of protein and drug alone, but rather drug concentration and dissociation constant. The halothane binding energies suggest K_d_ values between 6 and 16 mM. This yields a fractional occupancy of 1%–8% at 1 to 2 MAC, assuming the system is equilibrated. However, this does not account for the non-equilibrium state of biological systems, nor does it account for multiple affinity sites or sites of partial binding.

The existence of many sites with similar binding energies made it difficult to assign binding to any particular site(s). In fact, it is likely that anesthetics bind non-specifically to many of the predicted binding sites. Low persistence of a binding site does not necessarily indicate that a potential site is invalid. Rather, it implies a lack of favorable conditions for binding, since these sites are associated with greater overall conformational free energies of the protein system. However, anesthetic molecules may bind to low persistence sites, potentially with a greater binding energy than to higher persistence sites. In light of this, it is expected that at a constant anesthetic concentration, the sites that are most occupied are determined by the sum of the conformational energy differences, as reflected in persistence, and binding free energy differences.

A total of 32 binding sites (16 on α-tubulin, and 16 on β-tubulin) were predicted on a single tubulin dimer, which were independent of the dimer placement in MT geometry (see Table 2). Only one of the sites predicted on α-tubulin (site 23) persisted for more than 70% of the simulation, in fact lasting for the entirety of the simulation. Four sites predicted on β-tubulin (sites 1, 4, 7 and 21) lasted for more than 70% of the simulation, with two sites (4 and 21) lasting the entire simulation. An additional 8 sites were located at the intradimer α-β interface (see Table 3). These also did not depend on the placement of the dimer within the MT structure. Site 38 persisted in this region for 84.06% of the simulation, while sites 5 and 37 lasted for 100% of the time. Seven sites were dependent on the placement of the dimers within the MT lattice (see Table 3). One site was predicted at the dimer-dimer interface lasting for 88.05% of the simulation. The remaining 6 sites were located at the protofilament interfaces, either between α- or β-tubulins, however all of these persisted for less than 50% of the simulation. Reasonable anesthetic concentrations may thus alter only longitudinal, or intradimer interactions, but only at sufficiently high concentrations would lateral attraction be affected.

Binding energy estimates for halothane with the tubulin C-terminal tails are comparable to binding on the main protein body suggesting another mode of interaction. Larger binding energies exist for more compact conformations of the C-terminal tail. As such, due to the flexibility of the C-terminal tails, interaction with halothane may sequester the tail region, holding them in more compact forms, and preventing normal tail movements. This is of importance to the function of MTs as evidence indicates the C-termini play critical roles in regulating microtubule structure, function and interaction with MAPs [53]–[60]. Sequestration of the C-terminal tails by halothane into compact forms may indeed alter tubulin polymerization dynamics.

### Comparison to Known Drug Binding Sites

#### Taxol

Taxol is a MT-stabilizing agent that makes direct contact with a substantial number of the β-tubulin residues upon binding. The taxol-binding pocket is defined by the β-tubulin H1 helix (including βV23 and βD26), H6–H7 loop (including βL217 and βL219), H7 helix (including βH229, βL230, βA233 and βS236), M loop between strand S7 and helix H9 (including βF272, βP274, βL275, βT276, βS277 and βR278), and the S9–S10 loop (including βP360, βR369, βQ370 and βL371) [61]. Taxol acts to stabilize the M-loop increasing inter-protofilament interactions.

Of the key residues, anesthetic site 10 is within 5 Å of βL219, and site 42 is within 5 Å of βL217 and βP274. βP274 forms part of the hydrophobic pocket for the taxol 3′-phenyl group, while βL217 makes hydrophobic contact with the 2-phenyl ring of taxol, a component of taxol shown to be absolutely required for its activity [62]. βL219 also makes hydrophobic contact with the 2-phenyl ring.

Interaction of volatile anesthetics in this region may alter taxol binding resulting in loss of function. However, site 42 is negligibly persistent, only lasting for 0.80 % of the simulation, and site 10 persists minimally for ∼27% of the simulation. Therefore, this behavior would only be expected in cases of high anesthetic concentrations.

The tubulin polymerization experiment suggests that this is indeed the case. Tubulin in the presence of 10 µM of paclitaxel produced similar optical density curves over time in both the presence and absence of 40 mM concentration of halothane. Thus, halothane does not affect the interaction of taxol with tubulin consistent with the computational prediction.

#### Colchicine

Colchicine inhibits MT polymerization by binding to free tubulin dimers at the intradimer interface and interacting with both α- and β-tubulin residues. The colchicine site is mainly located within the β-tubulin, and is surrounded by residues βC241, the β-tubulin T7 loop, H8 helix (including βD251), S8 strand (including βV318), and S9 strand (including βK352), and interacts with residues αE71, αN101, and the α-tubulin T5 loop (including αV181) [63].

Steric hindrance between αV181 and colchicine prevents the α-tubulin subunit from occupying its normal position interfering with the straight conformation adopted by tubulin in protofilaments. The loss of this straight conformation prevents the tubulin M-loop from establishing lateral contacts between protofilament ends, leading to hindrance of MT dynamics and eventually MT depolymerization (or lack of MT polymerization from free tubulin). Additionally, binding of colchicine requires movement of the β-tubulin T7 loop and H8 helix to accommodate the drug molecule, and this movement also interferes with the α-β interaction.

Site 38 is within 5 Å of residue αV181, of the α-tubulin T5 loop, and the β-tubulin H8 helix and S8 strand, and persists for a considerable 84.06% of the simulation. Site 7 is located within 5 Å of residue βC241, and the β-tubulin T7 loop and S9 strand and persists for 78.88 %, while site 41 persists for 56.18% and is located within 5 Å of the β-tubulin H8 loop. Site 34 is located within 5 Å of the β-tubulin S8 and S9 strands including residue βK352, and site 46 is within 5 Å distance of the α-tubulin T5 loop, including residue αV181, and βK352 of the β-tubulin S9 strand, however they persist minimally for less than 20% of the simulation.

**Figure 7:**
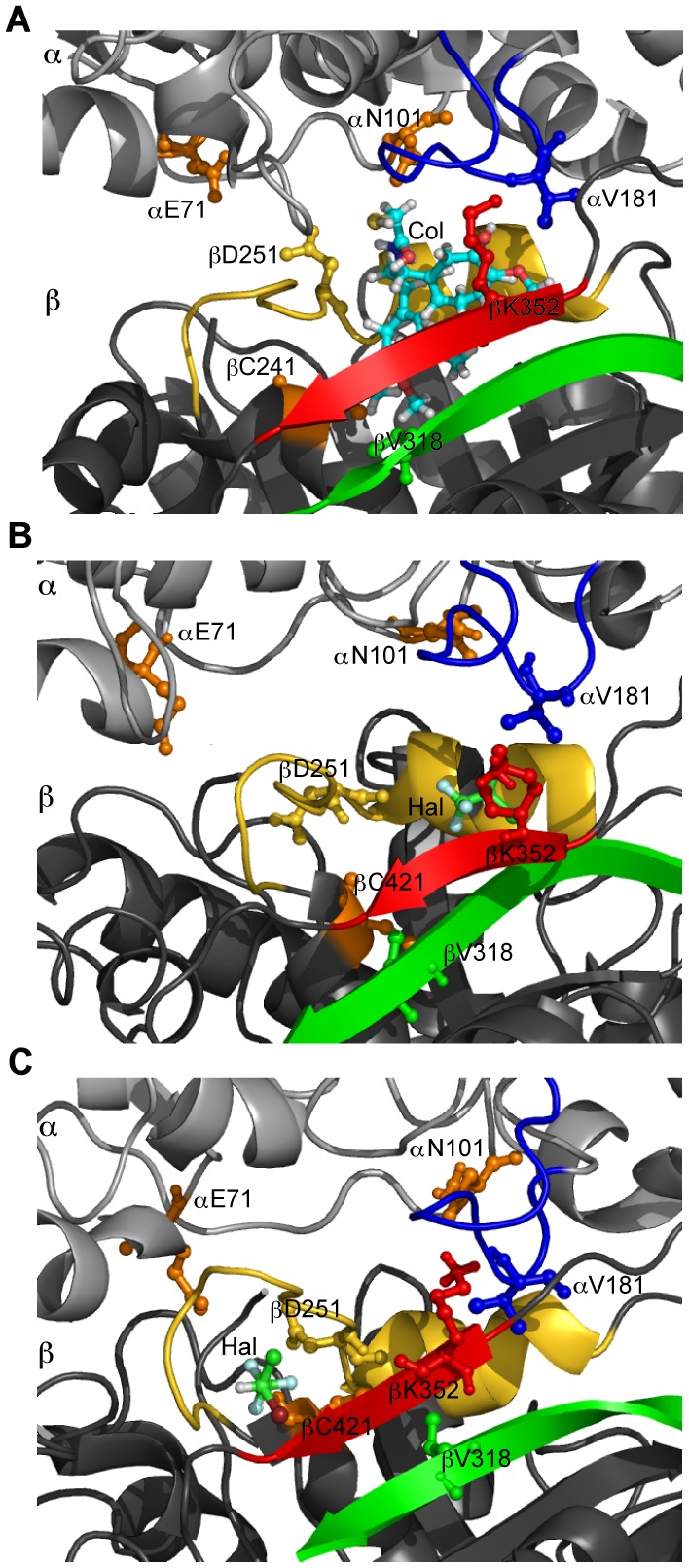
Halothane binding in the colchicine-binding pocket. Blue – loop αT5, Red - strand βS9, Yellow – loop βT7 and helix βH8, Green – strand βS8, Orange – αE71, αN101, and βC241. (A) Colchicine binding site. (B) Halothane binding site 38, -2.72 kcal/mol, surrounded helix βH8 and strand βS8. (C) Halothane binding site 7, -3.13 kcal/mol, within 3 Å of βC241 and surrounded by strand βS9 and loop βT7.

The preferred binding mode of halothane at site 38 was shown to occupy a space surrounded by helix βH8 and strand βS8, while the binding mode at site 7 is within 3 Å of βC241 and surrounded by helix βH8 and strand βS9 (see Figure 7). Van der Waals interaction of halothane with the βH8 helix would reduce the mobility of these residues preventing the accommodation of the colchicine molecule in this pocket. This is consistent with findings that report a reduction in colchicine binding to tubulin in the presence of halothane [64].

Interaction of volatile anesthetics with the β-tubulin T7 loop or H8 helix can reduce the mobility of these regions preventing the movement required to accommodate the colchicine molecule within tubulin. Additionally, interaction of volatile anesthetics with residue αV181 can hinder the movement of the α-tubulin T5 loop produced by the steric clash with colchicine. As these sites are persistent it is thus expected that volatile anesthetics would hinder the binding of colchicine. The effect of volatile anesthetics on the binding of colchicine derivatives must also be considered as it is known that colchicine derivatives substituted at the methoxy positions of ring A can be cross-linked with βC241 and anesthetics at site 7 may reduce this interaction.

#### Vinblastine

Vinblastine is one of the vinca alkaloids and is an inhibitor of tubulin polymerization. The primary binding site of vinblastine is located at the MT plus (growing) end towards the inner lumen of the MT and works by inhibiting longitudinal (dimer-dimer) contacts in a protofilament. The vinblastine binding-pocket is boxed by the α-tubulin T7 loop (including αL248 and αN249), H10 helix and S9 strand (including αL352), and the β-tubulin T5 loop (including βV177 and βD179), H6 helix carboxy-terminal turn (including βY210), and the loop region between helices H6 and H7 (including βF214) [65]. Vinblastine affecting these regions alters the longitudinal interface between tubulin dimers constraining them in a curved assembly to avoid steric clashes with the drug molecule. The curvature induces displacement of the M-loop on subsequent dimers weakening lateral interactions leading to reduced MT dynamics and eventual MT depolymerization.

Site 21, which persists for the entirety of the simulation, is within 5 Å of βY210 in the carboxy-terminal turn of helix H6. Site 39, which persists for 88.05% of the simulation, and site 1, which persists for 70.12% of the simulation, neighbor residue βD179. Site 1 is also adjacent to βV177. Interaction of volatile anesthetics with these residues is expected to alter vinblastine binding either through competitive binding, or hindrance of the mobility required to avoid steric clashes. Sites 19 and 20 are in the region of the α-tubulin H10 helix, and sites 17 and 42 are within the β-tubulin H6 helix and H6–H7 loop regions. However, these sites all exhibit low persistence.

The effect of volatile anesthetics on derivatives of vinorelbine, a vinblastine analogue modified on the D’ ring, should also be considered. Changes in these derivatives, which yield modified affinities and activities, occur in regions that interact with βY224 [66], a residue within distance of site 21.

**Figure 8:**
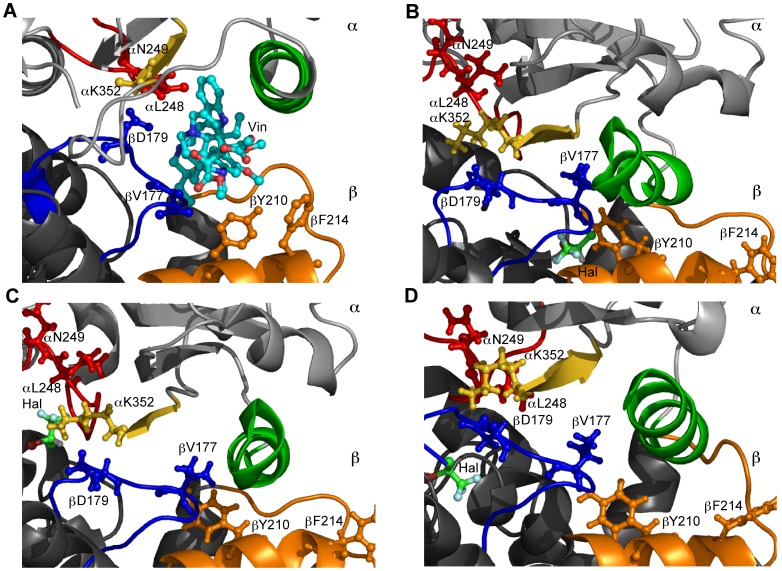
Halothane binding in the vinblastine-binding pocket. Red - loop αT7, Yellow – helix αH10, Green – strand αS9, Blue – loop βT5, Orange – helix βH6 and loop βH6-βH7. (A) Vinblastine binding site. (B) Halothane binding at site 21, −2.85 kcal/mol, within 2 Å of βY210 and surrounded by βH6 and βH7. (C) Halothane binding at site 39, −2.44 kcal/mol, within 5 Å of αK352 and βD179. (D) Halothane binding at site 1, −3.04 kcal/mol, within 3 Å of βD177 and surrounded by βT5.

Halothane binding in the vinblastine pocket is shown in Figure 8. The binding mode of halothane at site 21 is within 2 Å of βY210 and the molecule is surrounded by helices βH6 and βH7. Halothane binding at site 39 is within 5 Å of αK352 and βD179 and is cradled by the surrounding αT7 loop. Halothane at site 1 lies within 3 Å of βD177 and is surrounded on each side by loops βT5 and βT4. Due to the van der Waals interaction halothane can alter the mobility of these regions key to vinblastine binding potentially reducing vinblastine binding.

One point of interest is that vinblastine and colchicine are known to inhibit assembly at intermediate concentrations while inducing MT-to-macrotubule transformations at high concentrations [67]–[69]. Indeed it has been shown that halothane and other volatile anesthetics may induce the same aberrant forms of tubulin polymerization, albeit at extremely high concentrations [43], [44], [70]. Preferential binding of halothane to a less stable conformation of the vinblastine or colchicine cavity may result in destabilization of the longitudinal interaction resulting in an effect similar to that of these antimitotic drugs.

#### Peloruside A

The macrolide peloruside A is a MT-stabilizing agent that synergizes with taxoid drugs by acting at a unique site. The proposed peloruside A binding site is located on the exterior of the β-tubulin and is defined by the H9 helix (including βQ294), the H9–H9’ loop (including βD297 and βR308), the H9’ helix, the H9’-S9 loop, portions of strand S8, and the H10 helix (including βV335, βN339 and βY342) [71]. It is suggested that peloruside A stabilizes protofilament interactions by securing the α-tubulin T5 loop with the adjacent residues found in the β-tubulin H9–H9’ loop, the H9’ helix, and the H9’-S8 loop, although the exact mechanism is not known.

Site 4 contacts the H9 helix, the H9–H9’ loop, the H9’ helix and the H9’-S8 loop and persists for 100% of the simulation. Additionally site 37 and site 38 contact the H9’ helix and H9’-S8 loop, and persist for 100% and 84.06% of the simulation, respectively. While the exact mechanism by which peloruside A acts is unknown, it can be expected that volatile anesthetics in this binding- region would alter both drug binding, and the resulting conformational changes which yield its stabilizing effect at reasonable anesthetic concentrations.

Sites 9, 17, 30, 31, 34 and 42 are all within this region as well, however their persistence is low. At high anesthetic concentration it is expected that the multitude of potential sites in this region would alter the effects of peloruside A significantly.

#### Laulimalide

Laulimalide is a potent, macrolide MT-stabilizing agent that binds to the exterior of the MT on β-tubulin near the C-terminal E-hook. The binding site, which encompasses the proposed peloruside A pocket, is surrounded by the β-tubulin H9–H9’ loop (including βF296), H9’ helix (including βR308), H10 helix (including βV335) and the H10–S8 loop (including βN339 and βY342) [72]. Laulimalide does not appear to bind to the α-tubulin.

Upon laulimalide binding both βR308 and βY342 reorganize to generate a cation-π interaction, increasing the stabilization of the loops in this pocket. In this configuration βR306 establishes polar contacts with the oxygen of the dihydropyran, in the laulimalide side chain. The hydrophobic βV335 and βF296 residues also reorganize to align in parallel with the laulimalide macrolactone ring, with the movement of βF296 creating an entrance into a deep cavity in β-tubulin. Finally, the βN337 residue, which also defines this cavity, forms a hydrogen bond with the hydroxyl group at laulimalide C15.

As with peloruside A, Site 4, 37 and 38 persist in this region for a considerable portion of the simulation suggesting that volatile anesthetics binding in this region would alter both drug binding, and the resulting conformational changes. Sites 9, 17, 30, 31, 34 and 42, again, would be expected to play a role only at high anesthetic concentration. One key point of interest is that site 4 is within 5 Å of βF296. Reduced mobility of this residue, by interaction with an anesthetic molecule, is expected to inhibit binding of the laulimalide molecule through unfavorable alignment of βF296 with the laulimalide macrolactone ring and subsequent shrinkage of the deep cavity entrance. Additionally, even though laulimalide may possess a greater binding affinity for tubulin than the volatile anesthetics, small anesthetic molecules occupying the deep cavity region may be prevented from escaping by an impinging laulimalide molecule. This would result in a binding “stalemate” preventing proper laulimalide effects.

### Comparison to Nucleotide Binding Sites

#### Non-exchangeable GTP

α- and β- tubulin each bind one molecule of GTP (or GDP). GTP bound to the α-tubulin, at the N-site, is non-exchangeable and cannot be hydrolyzed to GDP. The N-site is located at the intra-dimer interface and is characterized by the α-tubulin H1 helix (including αG10, αQ11, αA12, αQ15, and αI16), T2 loop (including αD69, αL70 and αE71), H2 helix (including αV74), T3 loop (including αA99, αA100, and αN101), T4 loop (including αS140, αF141, αG142, αG143, αG144, αT145 and αG146), T5 loop (including αI171, αY172, αP173, αA174, αT179, αA180 and αE183), αN206 of the T6 loop, and H7 helix (including αY224, αL227, αN228 and αI231), as well as the β-tubulin T7 loop (including βL248 and βN249), βK254 of the H8 helix, and βK352 of the S9 strand [61].

Site 5, 23, 38 and 7 all persist for a significant portion of the simulation (100%, 100%, 74.06% and 78.88%, respectively), and all neighbor residues that directly interact with either GTP or the magnesium ion (Mg^2+^) required for the intradimer α-β stability. Sites 2, 24, 34, 35, and 46 are also in this same region, but persist for less than 21% of the simulation. Anesthetics at these sites may interfere with interactions between GTP, Mg^2+^, and tubulin. The effect of volatile anesthetics on GTP binding has not been studied for tubulin, but it has been found that volatile agents, at clinically relevant doses, have a direct effect on the conformation and stability of the GTP/Mg^2+^ bound state of some, but not all, GTP binding proteins [73]. This indicates the possibility of volatile anesthetics altering GTP dependent MT dynamics resulting in reduced polymerization, however further investigation is needed.

Also of note, site 5 borders residues αI171 and αT179, and site 38 is adjacent to residue αA180, which are all part of the sugar binding T5 loop. These residues are involved in longitudinal contacts between dimers, and the lateral, protofilament, interactions. This suggests a possible anesthetic induced MT depolymerization mechanism.

#### Exchangeable GTP

GTP bound to the β-tubulin, at the E-site, is exchangeable and can be hydrolyzed to GDP. GTP is required at the E-site in order for tubulin to polymerize, but is hydrolyzed to GDP upon polymerization. The result is a metastable MT structure stabilized by a so-called cap of remaining GTP-tubulin subunits. The loss of this cap results in rapid depolymerization.

The E-site is located at the inter-dimer interface and is characterized by the β-tubulin H1 helix (including βG10, βQ11, βC12, βQ15, and βI16), T2 loop (including βD69, βL70 and βE71), H2 helix (including βT74), T3 loop (including βA99, βG100, and βN101), T4 loop (including βS140, βL141, βG142, βG143, βG144, βT145 and βG146), T5 loop (including βV171, βV172, βP173, βS174, βD179, βT180 and βE183), βN206 of the T6 loop, and H7 helix (including βY224, βL227, βN228 and βV231), as well as the α-tubulin T7 loop (including αL248 and αN249), αE254 of the H8 helix, and αK352 of the S9 strand [61].

Sites 1, 21 and 39 all persist for a significant portion of the simulation (70.12%, 100%, and 88.05%, respectively), and all neighbor residues that directly interact with either GTP or the magnesium ion (Mg^2+^) in the non-hydrolyzed state, or with GDP in the hydrolyzed state. Sites 10 and 17 also make contact with key residues, but persist for less than 28% of the simulation. This indicates a potential for volatile anesthetics to affect GTP dependent polymerization dynamics as discussed above.

Similar to the N-site, sites 1, 21 and 39 neighbor residues in the T5 loop, potentially affecting lateral interactions between protofilaments. Additionally, site 21 encompasses βN206. Tubulin’s preference for GTP is the result of hydrogen bonding of the 2-exocyclic amino group in GTP to the hydroxyl groups of the N206 and N228 residues, and by hydrogen bonding of the 6-oxo group to the amino group of N206 [61]. Interactions of anesthetics with βN206 at the E-site could potentially reduce tubulin’s specificity for GTP hampering the stabilizing nature of the GTP cap resulting in increased depolymerization events.

### Comparison to MAP-binding Sites

MAPs bind to polymerized tubulin to regulate MT dynamics. The numerous MAPs identified carry out a wide range of functions dependent on the host tissue in which they are found. These functions include stabilizing and destabilizing MTs, guiding MT transport, cross-linking of MTs, and mediating interactions between MTs and other proteins or membranes. In the neuron MAP1A, MAP1B, MAP2 and tau are the most prominent MAPs, and all serve to stabilize MTs.

The exact binding location of these MAPs on tubulin is not known. However, it is known that MAP2 and tau share a sequence similarity in their MT binding repeats close to their carboxyl ends [74], [75]. The proline-glycine-glycine-glycine (PGGG) motifs, believed to form tight turns upon association with MTs, are analogous to a sequence in the S9–S10 loop of α-tubulin (residues T361–L368), which fills the equivalent of the taxol site in the α-tubulin stabilizing the M-loop conformation [76], [77]. It is believed that MAPs occupy the taxol-binding region in the β-tubulin securing the M-loop and stabilizing MTs in a manner similar to the action of taxol.

As discussed above, interactions of volatile anesthetics with MTs are not expected to alter the effect of taxol except at high anesthetic concentration. This can be extended to include MAPs acting by the same mechanism. The effect of volatile anesthetics on other MAP binding modes is unknown.

### Comparison to Post-translational Modification Sites

In animals, tubulin may undergo numerous post-translational modifications involved in regulating MT stability and the interaction with MAPs. The α-tubulin may undergo acetylation, tyrosinolation/detyrosinolation, deglutamylation, palimotoylation, polyglutamylation polyglycylation, and phosphorylation whereas the β-tubulin may only undergo the latter three [78]. The majority of these modifications, including tyrosinolation/detyrosinolation, deglutamylation, polyglutamylation, and polyglycylation, occur in the C-terminal region of tubulin.

Post-translational modifications require enzymes to interact with the C-terminal region, and it is expected that this process would require the tails to exhibit normal flexibility. The interaction of halothane with the C-terminal tails indicates an increase in binding energy with the number of residues interacting with the molecule. Halothane may thus serve to reduce the motion of these tail regions interfering with normal post-translation modification processes.

Acetylation of α-tubulin takes place at the ε-amino group of αK40. Palimotoylation occurs on α-tubulin C376. No predicted volatile anesthetic sites reside near these locations.

Phosphorylation takes place on both α- and β-tubulin at tyrosine residues, or serine/ threonine residues on after residue 307 [79]. Several predicted sites reside near serine or threonine residues in this region, or near tyrosine residues, but the majority of these sites persist for less than 50% of the simulation. However, site 38, persisting for 84.06% of the time, is within 5 Å of βT314. Additionally, site 21, which lasts for the entire simulation, is within 5 Å of both βY210 and βY224. This indicates a potential interference of volatile anesthetics with tubulin phosphorylation, yet the exact location of this modification is not known. This is of interest as tubulin phosphorylation has been suggested as a molecular mechanism for memory encoding [80], [81].

### Comparison to Zinc Binding Sites

In regards to Alzheimer’s Disease, zinc has been shown to promote the aggregation of β-amyloid, which then sequesters the metal making it largely unavailable, causing zinc dyshomeostasis in the vicinity of β-amyloid deposits [82]. While β-amyloid plaques are necessary to initiate the neurodegenerative process in AD, it is the NFTs that lead to neurodegeneration. Recently it has been argued that zinc sequestration by β-amyloid deposits deplete intra-neuronal zinc which drives formation of NFT, MT destabilization and associated neuronal degeneration [83]. The study, which investigates zinc binding to MTs and their component tubulin proteins, predicts 6 zinc-binding sites under the highest stringency.

Cysteine, histidine, aspartic acid and glutamic acid amino acid residues are key to zinc binding and account for ∼97% of all zinc-binding amino acids [84]. The 6 predicted binding sites and key residues involved include the site associated with zinc-induced tubulin sheet formation (including αH192, αE420 and αD424) [61], a site near the non-exchangeable GTP site, an uncharacterized site on the α-tubulin (including αH266 and αD431), a site near the colchicine binding site (including βC243, βC356 and βD357), and two uncharacterized sites on the β-tubulin.

Site 33 neighbors the αH266 and αD431 residues of the uncharacterized α-tubulin site, but persists for a mere 1.59% of the simulation, indicating that volatile anesthetic interactions with zinc bound at this site are not expected to play a significant role unless high anesthetic concentrations are used. Site 8, which persists for 51% of the simulation, is within 5 Å of αD424 of the zinc-induced tubulin sheet formation site. This site is only expected to play a role in tubulin polymerization at zinc concentrations well above physiological conditions, however the zinc-binding site near the colchicine site is expected to play a significant role in tubulin polymerization at physiological zinc concentrations [83]. Site 7, and 44 border on the colchicine zinc-binding site. Site 7, which persists for 78.88%, is near βC241, while site 44, which persists for 22.71%, is adjacent to βC356 and βD357. Due to their large dipole moments, the presence of a volatile anesthetic in these regions is expected to interfere with van der Waals forces required for the residue coordination necessary for zinc binding. Should this occur volatile anesthetics would temporarily inhibit zinc binding, reducing the tubulin polymerization rate, resulting in a net loss of polymerized MTs, similar to the effect caused by β-amyloid zinc sequestration [83]. This could potentially cause memory impairment effects.

### Comparison to Aromatic Amino Acids

While the mechanism of general anesthetic action responsible for erasing conscious awareness is still under debate, quantum mobility theory posits that anesthetics act by inhibiting signal propagation between the aromatic amino acids of tubulin [85]. Aromatic amino acids include tyrosine, tryptophan, phenylalanine and histidine, of which tubulin possesses 35, 8, 43 and 22, respectively. Numerous sites are near tyrosine residues (sites 11, 12, 16, 21, 22, 24, 32 and 33), phenylalanine residues (sites 4, 15, 16, 24, 25, 28, 32, 41, and 45), and histidine residues (sites 14, 22, 25, 26, 33 and 36) with some lasting for the entire simulation. Of these, tryptophan is posited to play a prominent role in quantum mobility theory. The closest predicted site to a tryptophan residue is site 37, persisting for 100% of the simulation, which is within 5.3 Å of βW346. This is consistent with the predictions of quantum mobility theory.

### Experimental Validation

Numerous historical studies give evidence to the interaction between halothane and tubulin polymerization. The earliest studies showed concentrations of 2–2.8 % halothane in air by volume (∼0.1 mM) yielded retraction of MT based axopods suggesting MT depolymerization [41], [42]. Consistent results were found with larger concentrations of halothane. At 10 mM halothane, MTs were shown to decrease in length and density [44], and MT based flagellar structures were shortened [46], and 20 mM halothane likewise decreased MT density [45], [86]. However, other investigations showed concentrations between 3 and 10 mM increased the number of MTs per area [44], [86], [87]. Other evidence indicates that halothane concentrations of 10 mM can produce aberrant MT formations, including ribbon structures and macrotubules [43], [44], [88].

This range of behaviors can be attributed to differing polymerization protocols. Tubulin polymerization is affected by many factors including pH, temperature and ionic concentrations. Similar variations in behavior were observed when first characterizing the effect of zinc on tubulin polymerization [89]. By varying buffer conditions and the ratio of tubulin to zinc, polymerization ranged from normal to aberrant, with various intermediate stages.

It is important to note that optical density measurements were not capable of quantifying polymerization of these zinc induced aberrant tubulin structures [89]. Optical density measurements also cannot detect the aberrant assembly of tubulin induced by the MT destabilizers cryptophycin 1 [90], [91], [92] and hemiasterlin [90]. The kinetics of MT assembly are often studied by optical density measurements since the geometry of long thin rods is such that absorption data is a function of the total mass of the assembled protein. As such, without knowledge of the shape of tubulin aggregates it would be dangerous to interpret absorption data quantitatively.

In the present study, a halothane concentration of 40 mM was used to ensure saturation of tubulin by halothane. The optical density measurements show that in the presence of both 10 µM paclitaxel and 40 mM halothane the optical density curve is comparable to that of paclitaxel alone within standard error. This suggests that halothane has no effect on the interaction between paclitaxel and tubulin, a result consistent with the computational predictions presented. In the presence of 40 mM halothane alone the optical density curve resembles the control, however the standard error indicates difference between the curves with a small increase in the optical density. While this seems to indicate that halothane does little to modulate tubulin assembly, previous evidence of altered tubulin structures and the inability of optical density to quantify such changes suggest that this may not be the case. Further investigation is clearly required.

### Summary

Anesthetic binding to tubulin may be important for the mechanism of anesthetic action, and also for anesthetic side effects related to post-operative cognitive dysfunction and/or exacerbation of neurodegenerative diseases. The size of the tubulin macromolecule, its numerous preexisting non-polar, hydrophobic cavities, and the generally weak binding of volatile anesthetics hinder the experimental investigation of this molecular mechanism of interaction. For this reason, it is an important problem to be able to predict computationally the binding of anesthetics to tubulin, and of small molecules to proteins in general, although this is a challenge since binding is often nonspecific, with multiple binding sites being filled. Thus, we have used a combination of computational methods including molecular dynamics simulations, surface geometry based anesthetic binding site prediction, focused and blind docking to identify putative volatile anesthetic binding sites on, and in, the tubulin protein. We hope that this work will enable future experiments to resolve the necessary ambiguity in our computational results.

Multiple binding sites were found on the tubulin protein, but the availability of these sites for anesthetic binding was found to vary greatly. Since most binding energies were quite close in value, it is expected that those with lowest persistence will be filled only at large anesthetic concentrations.

Since the binding energy of volatile anesthetics in protein hydrophobic pockets is generally small, the potential for anesthetic molecules to bind by inducing fits through changes in protein conformation is extremely low. It is more likely that these molecules bind in preexisting non-polar, hydrophobic cavities. In this case destabilization of a protein, or protein structure, may result either from preferential binding of the anesthetic to a less stable conformation of the cavity, or the disruption of allosteric changes at protein interfaces.

We found that favorable thermodynamic conditions for the binding of anesthetics to tubulin result from van der Waals interactions. The nine sites predicted to persist for greater than 70% of the 5 ns simulation were located in the binding-pockets for colchicine, vinblastine, peloruside A, laulimalide, GTP, and GDP. These sites all reside in regions of either intradimer, or longitudinal interaction, indicating that at reasonable concentrations anesthetics do not alter lateral interactions. In fact, we do not predict strong binding in the taxol-binding site, which has been implicated in lateral interactions. This was also confirmed by experimental validation within the present investigation.

Our findings suggest that modification of intradimer and longitudinal interactions may be the general mode of MT destabilization by volatile anesthetics. This is consistent with observations that anesthetics are weak destabilizers of MTs under normal conditions. Steric hindrance caused by the antimitotics vinblastine and colchicine result in tubulin being constrained to a curved conformation preventing MT polymerization, and promoting the formation of macrotubules. At high concentrations anesthetics are shown to have the same effect, indicating the possibility of a similar mechanism, thus highlighting these putative binding sites. Indeed, our prediction of the interference of halothane with the binding of colchicine to tubulin has already been confirmed by an experimental study [64].

While anesthetic binding in protein hydrophobic pockets is the expected mode of interaction, we found comparable binding energy estimates between the C-tails and the tubulin body indicating an interaction between halothane and the tubulin C-termini. Our results show an increase in binding energy with more surrounding residues of the tail suggesting that halothane may sequester the C-termini into compact forms. However, solvent effects are expected to play a prominent role in this interaction and docking alone cannot provide the detailed energy evaluations needed to investigate this mechanism. The role of C-termini in normal MT dynamics, including polymerization, post-translational modification, and interaction with MAPs, marks this potential interaction as one of interest.

The interaction of volatile anesthetics, including halothane, with tubulin and MTs is of great interest both for the mechanism of action of anesthetic gases, and for post-operative cognitive dysfunction and neurodegenerative diseases that present with dysfunctional neuronal cytoskeletons [31]. Neurons are unique as they are non-dividing cells, and thus their MTs are not required to repeatedly assemble and disassemble as in mitotic spindles. Part of this is due to the neuronal cytoskeleton, which is highly architectured and relatively stable. This stability both serves to prevent division and maintain neuron morphology, which is essential to overall neural function. Halothane has been shown to bind to tubulin, alter tubulin polymerization, and disrupt polymerized MTs [40]–[47]. This suggests a potential cause of anesthetic induced exacerbation of neurodegenerative disorders. In regards to postoperative cognitive dysfunction, our results suggest that anesthetics may not alter MAP binding directly to release tau and increase NFT formation. However, altered MT polymerization, either through the modification of intradimer/longitudinal contacts, C-terminal tail dynamics, or through effects on cofactors relevant to polymerization, such as GTP/GDP or zinc, may result in either net loss of polymerized MTs, or MTs polymerized into aberrant forms. Due to the overall reduced number of normal MTs, an excess amount of free tau could arise. This free tau would be vulnerable to hyper-phosphorylation leading to an increase in NFT formation. Further investigation would be required to test these hypotheses.

The role of MTs in cell division marks tubulin as a prominent chemotherapeutic target. As such there exists the possibility of adverse drug reactions between volatile anesthetics, cancerous cell lines and antimitotic agents [93]. Thus, our results may not only shed light on the role of anesthetics in non-dividing neuron cells, but also on dividing cells, especially cancer cells, due to the prominent role of tubulin based MTs in these two classes of cells. Due to the potential involvement in post-operative cognitive dysfunction, and the potential adverse drug reactions between anesthesia and chemotherapy the volatile anesthetic-tubulin interaction warrants further investigation.

## Methods

### Molecular Dynamics (MD) Simulations

#### Tubulin body

Human tubulin isotypes TUBA4A and TUBB, being the most prevalent forms in human tissues [94] were modeled according to previous homology methods [95], [96]. Initial set-up was performed in the leap and antechamber modules of AMBER9 [97]–[99] using the AMBER03 force field. To obtain globally minimized structures fifteen high temperature implicitly-solvated MT dimers were generated at a temperature of 5,000 K then slowly cooled to a target temperature of 300 K, using steps of 1,000 K over periods of 400 ps, then using a 0.8-power law cooling-schedule for temperatures below 2000 K. One of the resulting structures was chosen on which to perform MD simulations.

MD simulation included two adjacent protofilaments composed of two tubulin heterodimers. Tubulin dimers were placed in a configuration consistent with MT geometry [100]. Periodic boundary conditions were used to model the system as two periodic protofilaments. The protofilaments were aligned with their axis parallel to a periodic box of the length of one tubulin dimer, 81.2 Å. These were then relaxed for 300 steps without solvent, and another 300 steps with implicit solvent. Afterwards the structures were heated to 400 K, and then cooled to 300 K over a period of 400 ps to obtain further energy minimization.

Na^+^ ions were added to neutralize the system, and following this 80 Na^+^ and 80 Cl^−^ ions were added to bring the ionic concentration to 100 mM. The system was placed in a periodic box with dimensions 81.2 Å by 107.5 Å by 152.5 Å, filled with pre-equilibrated explicit TIP3P water using the leap module of AMBER9 [97]–[99].

To relieve steric clashes with water the structure was further energy minimized in GROMACS 3.3.2 [101]–[103] using 5000 steps of steepest descent, followed by 1000 steps of conjugate gradient minimization. Following equilibration for 5 ns with a time step of 2 fs, MD simulation, with periodic boundary conditions at 300 K and constant pressure was performed over a period of another 5 ns with the same time step. The SHAKE algorithm [104] was used to constrain bonds involving hydrogen in all simulations and a non-bonded cutoff of 12 Å was used.

#### Tubulin C-terminal tails

The 1JFF [61] model of tubulin was repaired with the missing residues of 1TUB [105]. The repaired 1JFF preparation was performed in the leap module of AMBER9 [97]–[99] using the AMBER99SB force field. The structure was explicitly solvated with TIP3P water in a 25 Å box extending from the protein surface. Thirty-five Na^+^ ions were added to neutralize the protein followed by the addition of 107 Na^+^ and Cl^–^ ions to bring the salt concentration to 100 mM. The structure was then energy minimized with a conjugate gradient method using NAMD [106] over 40,000 time steps.

Models of human tubulin isotypes TUBA1A, TUBA1C, TUBA4A, TUBB, TUBB2A /TUBB2B, TUBB2C, TUBB3, and TUBB4 were generated using MODELLER [107] 9v6 with the minimized, repaired 1JFF structure as a template, to produce 50 distinct conformations of each tubulin C-terminal tail.

### Volatile Anesthetic Binding Site Prediction

Trajectories from the 5 ns MD simulation were clustered into dominant conformations of the protein body using the g_cluster utility of the GROMACS [101]–[103] 3.3.2 program package with the single linkage method with 1 Å RMSD similarity cut-off comparing positions of all atoms, with C-terminal tails excluded. The average structures of the resultant clusters were subjected to further analysis. Percent simulation (% Simulation) of the average structures were calculated as the ratio between the number of timestep snapshots belonging a given cluster divided by the total number of timesteps in the MD simulation. Binding sites on the clusters were predicted using the surface geometry program PASS [51] modified specifically for volatile anesthetics [49]. The PASS [51] program was run on both the tubulin dimers in the protofilament conformation, as well as on each dimer separately to determine if MT geometry affected the prediction of a site.

Predicted binding sites between clusters were grouped via a Density Based Spatial Clustering of Applications with Noise (DBSCAN) [52] method with a minimum group size of 1 predicted site, and nearest neighbor distance of 5 Å. The center of each group was taken as the average position of all predicted sites within a given group. Not all predicted sites were found on all dominant conformations. Persistence of each site was determined by taking the sum of the simulation percentages for each of the dominant conformations on which the site was predicted.

### Halothane Docking

#### Tubulin body

Focused docking runs were performed for halothane at all predicted sites for the middle structures of all clusters of tubulin. Halothane geometry was parameterized according to an *ab initio* structure calculation [108]. Docking was performed via AUTODOCK [109] 4.0 using a slow focused docking protocol [110], and box size of 10 Å per and grid spacing of 0.375 Å. Binding poses were clustered with a RMSD of 2 Å.

#### C-terminal tails

Blind docking runs were performed for halothane against each of the 50 tail conformations for each tubulin isotype. Halothane, as parameterized above, was docked against the C-terminal tails via AUTODOCK [109] 4.0 using a blind docking protocol [111], [112]. Box sizes were adjusted to accommodate the C-terminal tail conformation with constant grid spacing of 0.375 Å. Binding poses were clustered with a RMSD of 2 Å.

All images were created in PyMOL 0.99rc6 [113].

### Microtubule Polymerization Assay

All protein and reagents required for the assay were purchased from Cytoskeleton Inc. (Denver, CO). 97% pure lyophilized bovine brain tubulin reconstituted in de-ionized water (10 mg/ml) was used. 200 µl aliquots were snap frozen in liquid nitrogen to maintain maximum protein functionality. Assays were carried out in a 96 well plate. Plates were pre-warmed at 37°C for 30 min prior to experiment. Using a 95% ethyl alcohol stock solution halothane (2-Bromo-2-chloro-1,1,1-trifluoroethane) (Sigma Aldrich, St. Louis, MO) and paclitaxel (Hospira, Lake Forest, Ill) were prepared to 11 times final concentration in General Tubulin Buffer (80 mM PIPES, MgCl_2_, 0.5 mM EGTA, pH 6.9). The alcohol concentration of the control was adjusted accordingly. 10 µl of halothane concentration, General Tubulin Buffer (control), paclitaxel and paclitaxel with halothane were preheated in separate wells of 96 well plate for 2 min. Immediately before running an assay tubulin was thawed in 37°C water bath and put on ice. Tubulin protein was diluted in 450 µl of ice cold Tubulin Polymerization Buffer (80 mM PIPES, 2 mM MgCl_2_, 0.5 mM EGTA, pH 6.9, 1 mM GTP 10.2% glycerol). 100 µl of tubulin and polymerization buffer solution were added to each well containing 10 µl for a final volume of 110 µl. Assay were run for 1 hour at 37°C, with absorbance measurements taken every 30 s at 340 nm using a SPECTROmax 190 (Molecular Devices, Sunnyvale, CA) plate reader and collected using SOFTmax Pro version 4.0 software.
